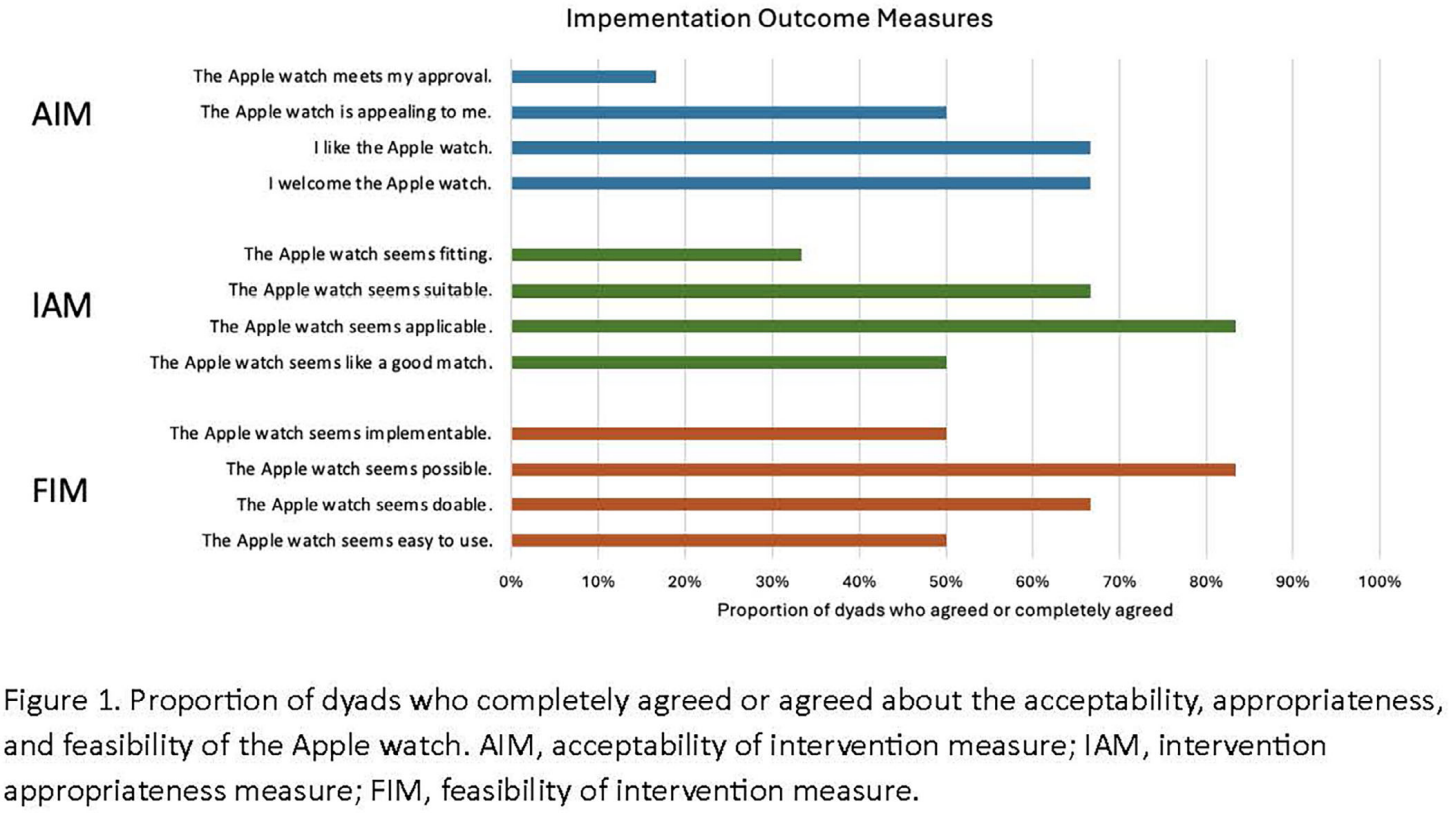# Acceptability, Feasibility, and Appropriateness of wearable technology in Lewy body dementia

**DOI:** 10.1002/alz70858_104487

**Published:** 2025-12-26

**Authors:** Bhavana Patel, Stephanie Staras, Todd Manini, Melissa J. Armstrong

**Affiliations:** ^1^ University of Florida, Gainesville, FL, USA

## Abstract

**Background:**

Wearable technology can provide valuable information about everyday activities of those living with Lewy body dementia (LBD) and their care partners. Nonetheless, there is a gap in the potential use of wearable technology and the extent to which it is currently utilized in those living with dementia. Evaluation of key implementation outcomes for wearable technology in this population can inform its use in future studies.

**Method:**

Implementation outcomes of acceptability, appropriateness, feasibility and usability of Apple watches were assessed amongst individuals with LBD and their care partners (i.e. dyads). Participants were mailed an Apple watch and asked to wear the watches for 2 weeks during hours of wakefulness with charging at night. Dyads completed questionnaires via a REDCap survey including: Acceptability of Intervention Measure (AIM), Intervention Appropriateness Measure (IAM), Feasibility of Intervention Measure (FIM), and System Usability scale (SUS). The “intervention” was the Apple Watch. The data was analyzed using descriptive statistical methods.

**Result:**

Between June 2023 and December 2023, six individuals with LBD and their care partners were enrolled (12 participants). Mean age of individuals with LBD was 71.3 years and care partners 67.5 years. Diagnoses included dementia with Lewy bodies (66.7%) and Parkinson disease dementia (33.3%), those with dementia identified as male with 66.7% having a disease duration of less than 4 years. All dyads completed the four questionnaires. Average scores (SD, range) for acceptability, appropriateness, and feasibility were 14 (2.28, 12‐18), 14.17 (2.23, 10‐16), and 13.83 (3.31, 8‐17) out of 20. Higher scores indicate greater levels of acceptance, appropriateness, and feasibility. The mean SUS score was 60.42 (SD: 7.97, 47.5‐67.5), where scores above 68 are considered acceptable. Three care partners experienced connectivity issues, which in one case resolved with restarting the watch however others were sent a new watch.

**Conclusion:**

The Apple watch was determined to be moderately acceptable, appropriate and feasible amongst individuals with LBD and their care partners. Usability was marginally acceptable. While studies with wearable devices hold promise, more feasibility testing is needed before their use as a primary outcome measure. Future studies should address implementation barriers and their solutions.